# The *Holobiont Blindspot*: Relating Host-Microbiome Interactions to Cognitive Biases and the Concept of the “*Umwelt*”

**DOI:** 10.3389/fpsyg.2020.591071

**Published:** 2020-11-16

**Authors:** Jake M. Robinson, Ross Cameron

**Affiliations:** ^1^Department of Landscape Architecture, The University of Sheffield, Sheffield, United Kingdom; ^2^In vivo Planetary Health, Worldwide Universities Network (WUN), West New York, NJ, United States; ^3^The Healthy Urban Microbiome Initiative (HUMI), Australia

**Keywords:** *Umwelt*, cognition, microbiome, system one thinking, *Holobiont Blindspot*, cognitive bias

## Abstract

Cognitive biases can lead to misinterpretations of human and non-human biology and behavior. The concept of the *Umwelt* describes phylogenetic contrasts in the sensory realms of different species and has important implications for evolutionary studies of cognition (including biases) and social behavior. It has recently been suggested that the microbiome (the diverse network of microorganisms in a given environment, including those within a host organism such as humans) has an influential role in host behavior and health. In this paper, we discuss the host’s microbiome in relation to cognitive biases and the concept of the *Umwelt*. Failing to consider the role of host–microbiome (collectively termed a “*holobiont”*) interactions in a given behavior, may underpin a potentially important cognitive bias – which we refer to as the *Holobiont Blindspot*. We also suggest that microbially mediated behavioral responses could augment our understanding of the *Umwelt*. For example, the potential role of the microbiome in perception and action could be an important component of the system that gives rise to the *Umwelt*. We also discuss whether microbial symbionts could be considered in System 1 thinking – that is, decisions driven by perception, intuition and associative memory. Recognizing *Holobiont Blindspots* and considering the microbiome as a key factor in the *Umwelt* and System 1 thinking has the potential to advance studies of cognition. Furthermore, investigating *Holobiont Blindspots* could have important implications for our understanding of social behaviors and mental health. Indeed, the way we think about *how* we think may need to be revisited.

## Introduction

It is well established that humans are prone to making systematic cognitive errors or “biases” – for example, the susceptibility to overestimate how much one understands about the world (Kahneman et al., 1998; Barton et al., 2016). Some authors (particularly those working within western scientific frameworks) have suggested that anthropomorphism – the heuristic act of attributing human-centric phenotypes to both non-human animals and inanimate phenomena – can lead to misunderstandings of non-human biological processes and behaviors (Burghardt, 2004; Farina, 2012; Bueno-Guerra, 2018). Furthermore, the hierarchical view of nature that positions humans as the pinnacle of species is yet another cognitive bias that may inhibit our understanding and appreciation of the complex interrelated ecologies of biology and behavior. It should, however, be acknowledged that many Indigenous societies view humans and the rest of nature as a complex web of interconnected subjects (and not discrete, hierarchical objects) (Gratani et al., 2016; De Castro, 2019; Robinson et al., 2020, Manuscript In Review).

The concept of the *Umwelt* was first coined by Jakob Von Uexküll in the early 20th century to describe phylogenetic contrasts in the sensory realms of different species, and the species-specific interactions that occur between the brain, the body and the environment (Von Uexküll et al., 1899; Von Uexküll, 1934/1957; Partan and Marler, 2002). Historically, the *Umwelt* was divided into the *Merkwelt* (perceptual world) and the *Wirkwelt* (effector/action world) to define an animal’s sensory unit, from perception to behavior. However, Bueno-Guerra (2018) recently proposed a broadening of the *Umwelt* concept to include the social sphere or the *Sozialwelt*. An important justification for this proposal was that social dynamics can profoundly influence perception and action. Moreover, transferring the human phenotype of “cooperative bonding” to their chimpanzee *Pan troglodytes* subjects, led to delusive generalizations in social behaviors (including inconsistent results in task solving with cooperative set-ups) i.e., evolutionary behavioral pathways may not be identical in other species.

In recent years, microbial ecology has seen a rapid expansion in knowledge – attributed in part, to technological advances such as high-throughput DNA sequencing and streamlined bioinformatics (the science of collecting and analyzing complex biological data) (Wooley and Ye, 2010; Stres and Kronegger, 2019). It has recently been suggested that the microbiome – the diverse network of microorganisms in a given environment – has an influential role in the behavior and health of humans and non-human organisms (Rook, 2013; Cryan et al., 2019; Sherwin et al., 2019). Indeed, microorganisms have recently been implicated in host behavioral manipulation through the olfactory system, the microbiota-gut-brain axis, and other biochemical pathways (Davidson et al., 2020; O’Donnell et al., 2020; Robinson and Breed, 2020). Furthermore, it is thought that exposure to the environmental microbiome plays an essential role in “educating” and regulating innate and adaptive immunity (e.g., via modulation of regulatory T cells), and microorganisms are known to provide a range of functional, physiological roles (Rook, 2013; Rook et al., 2014; Prescott et al., 2017; Chen et al., 2020).

In this perspective article, we discuss host-microbiome interactions in relation to cognitive biases and the concept of the *Umwelt*. We suggest that microbially mediated host behavioral phenotypes could provide the basis for another conceptual augmentation of the *Umwelt*, that is, to include explicit considerations for the microbiome in the realms of perception and action. Failing to consider the role of interactions between the host and their microbiome (collectively termed a “*holobiont”*) in a given behavior could underpin a potentially important cognitive bias which we refer to as the *Holobiont Blindspot*. This bias could lead to misinterpretations and delusive generalizations in animal (including humans) and non-animal behavioral studies. This is important from a third-person perspective (e.g., the researcher studying another organism or population). However, we also discuss whether microbial symbionts could have an influence from a first-person perspective (integral to the concept of the *Umwelt*) and in the dimension of System 1 thinking – that is, decisions driven by perception, intuition and associative memory, as popularized by Daniel Kahneman (Kahneman, 2001). If this is the case, there could be important social ramifications, and the concepts of perception and intuition may need to be revisited.

Recognizing the *Holobiont Blindspot*, and considering the microbiome as a key component of system that gives rise to the *Umwelt* and Systems 1 thinking, has the potential to advance studies of cognition and social behavior. Moreover, investigating these concepts could have important social ramifications by restructuring the way we interpret and empathize with social behaviors, and potentially how we understand mental health conditions.

## The *Holobiont Blindspot* and the *Umwelt*

Growing evidence suggests that the microbiome can have a considerable influence on the behavior of humans and non-human organisms (Farzi et al., 2019; Huang et al., 2019; Ezra-Nevo et al., 2020). Although the mechanisms of microbially mediated host behavioral responses are not fully understood, several biochemical pathways have been proposed. One pathway that has received considerable attention is the microbiota-gut-brain axis (Cryan et al., 2019; Lyte et al., 2020). This refers to the bidirectional communication system linking the central and enteric nervous system to the microorganisms in the gut via the vagus nerve (Ueno and Nakazato, 2016; Breit et al., 2018). Microorganisms in the gut produce an array of metabolic by-products that can stimulate peptide hormone secretion and directly activate the vagus afferents connecting the gut to the brain (Lach et al., 2018; Fülling et al., 2019). Consequently, it has been suggested that microorganisms can metaphorically “hijack” the gut-brain communication highway and influence a range of neuronal processes that result in behavioral responses (Vuong et al., 2017; Davidson et al., 2018). Gut microorganisms can also synthesize compounds such as serotonin (5-hydroxytryptamine), acetylcholine, and peptidoglycan which can penetrate the blood-brain barrier via the systemic circulatory system (Petra et al., 2015; Logsdon et al., 2018; Cryan et al., 2019).

A recent animal study demonstrated that gut bacteria can mimic thefunctions of cognate host receptor molecules to override host sensory decisions (O’Donnell et al., 2020). In this study, a commensal gut bacterium *Providencia* sp., produced a neuromodulator called tyramine. This compound is thought to act upon the host’s olfactory system, modulating aversive responses to certain odors. This process potentially drives mutually beneficial food decisions – i.e., the host is manipulated into choosing a food source that benefits both the animal host and the commensal bacteria.

This study is only one of several recent animal studies demonstrating modulation of host behavior by commensal bacteria. For example, the bacteria *Acetobacter pomorum* and *Lactobacillus* sp., have been shown to work synergistically to manipulate host feeding decisions in *Drosophila melanogaster* (Leitão-Gonçalves et al., 2017; Pasquaretta et al., 2018). Other *D. melanogaster* studies support the notion of behavioral manipulation via olfactory pathways – e.g., individuals can be attracted to compounds secreted by *Saccharomyces cerevisiae* and *Lactobacillus plantarum* but repelled by those from *Acetobacter malorum* (Qiao et al., 2019). Moreover, microorganisms are thought to trigger transcriptional olfactory responses in mice *Mus* sp., and zebrafish *Danio rerio* (Casadei et al., 2019; Cryan et al., 2019). Host sociability and breeding can also be influenced by the microbiome through the mediation of behavioral responses that influence inter-host transmission (Stilling et al., 2014; Wong et al., 2015; Shropshire and Bordenstein, 2016; Sherwin et al., 2019; Simon et al., 2019).

The intricate relationships between host and commensal microorganisms can be framed from a “hologenomic” perspective. A holobiont, a term first coined by Margulis (1990) is defined as a “*biomolecular network composed of the host plus its associated microbes [.], and their collective genomes forge a hologenome”* (Bordenstein and Theis, 2015).

It is important to acknowledge that the debate is ongoing as to how the hologenome concept of evolution may unfold. For example, an important criticism of this concept is that more evidence is needed to support the notion of vertical transmission of microbiota (from generation to generation) (Robinson and Breed, 2020). However, as Rosenberg and Zilber-Rosenberg (2020) point out, there is some evidence to support this concept. For example, human individuals can retain the same ancestral *Helicobacter pylori* strains, even after migrating to different localities (Achtman et al., 1999; Falush et al., 2003), and other corroborating studies were put forward by Rosenberg and Zilber-Rosenberg (2020) (e.g., Ochman et al., 2010; Goodrich et al., 2016; Moeller et al., 2016). Nonetheless, perhaps a more compelling argument for the hologenomic evolutionary process and its associated behavioral implications, arrives from the notion of *functional associations*. For example, it is likely that evolution has favored host-microbiome functional associations that precisely reproduce the biochemical networks that give rise to host behaviors across generations (Doolittle and Booth, 2017). Indeed, Suárez (2020) and Suárez and Triviño (2020) argue that in terms of defining the holobiont as an evolutionary unit, less emphasis should be placed on the microbiome’s lineages or taxa, and more on its functional traits (encoded by the organisms’ genes) – referred to as the *stability of traits* concept.

Whilst the precise evolutionary mechanisms still need to be unraveled, one element is clear: the microbiome’s functional traits can have a considerable influence on host perception of stimuli (*Merkwelt*) via sensory influences (e.g., olfactory processes), and subsequent behavioral responses or decision-making (*Wirkwelt*). Therefore, this concept could have important implications for evolutionary studies of cognition and may potentially present a cognitive bias if not considered. Here, we propose the *Holobiont Blindspot* to describe this potential cognitive bias. This cognitive bias – also known as a “blindspot” – could conceivably lead to misinterpretations and delusive generalizations as demonstrated by Bueno-Guerra’s (2018) *Sozialwelt*. Indeed, understanding the full sensory spectrum that an animal can perceive (e.g., one element being microbially derived odors), along with the unique drivers of perception and response (e.g., those functionally mimicked by commensal microorganisms) could aid in the selection of appropriate controls and relevant stimuli in behavioral studies. Just as a cognitive bias can manifest through the attribution of human-centric phenotypes to non-human animals, treating holobionts as individual subjects divorced from any cognitive influence via symbiotic interactions could also be viewed in this manner. It is also important to note here that plants and even microbes can themselves be holobionts. For example, this was articulated in a recent book, the Entangled Life (Sheldrake, 2020), with the following paraphrased passage:

*“I attended a conference in Panama on tropical microbes. Someone got up to talk about a group of plants that produced a certain group of chemicals in their leaves. Until recently, the chemicals had been thought of as a defining characteristic of that group of plants. However, it transpired that the chemicals were actually made by fungi that lived in the leaves of the plants. Our idea of the plants had to be redrawn. Another researcher interjected, suggesting that it may not be the fungi living inside the leaf that produced these chemicals, but the bacteria living inside the fungi. The notion of the individual had deepened and expanded beyond recognition. To talk about individuals made no sense anymore”* (*Sheldrake, 2020, p. 18*).

Whilst the idea of a *Holobiont Blindspot* was initially conceived with the third-person view in mind (e.g., the researcher studying the holobiont), we can also consider the *Holobiont Blindspot* from the first-person perspective – which is considered integral to the concept of the *Umwelt* (Baggs and Chemero, 2019). Indeed, in the human dimension the *Holobiont Blindspot* can be positioned in the realm of System 1 thinking. This refers to a conceptual branch of cognition characterized by “fast and automatic thinking” – popularized by Daniel Kahneman (Kahneman, 2001; Moran, 2012). It is important to note that running contra to System 1 thinking is System 2 thinking – a term used to describe the controlled and deliberate mode of thought (Rottenstreich et al., 2007). However, we find the former to be more relevant to the concepts and scope of this work.

**FIGURE 1:**
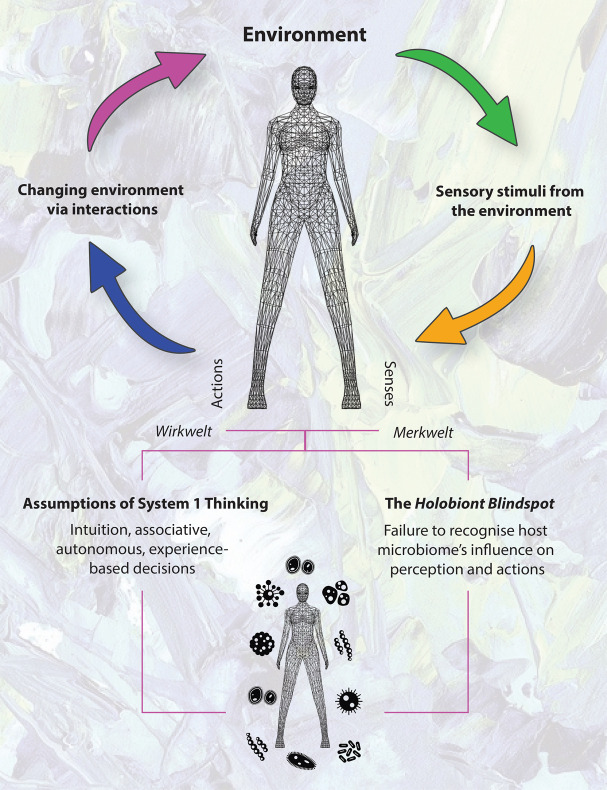
The perception-action cycle and assumptions of System 1 Thinking. Failure to recognize potential microbiome influences in perception and action is the *Holobiont Blindspot*.

Indeed, potential cognitive biases could occur if we assume a System 1-based response in the perception-action cycle (a central principle of the *Umwelt*, also known as the “functional loop”) as being purely the result of human intuition and/or associative memory, when it could conceivably be a microbially mediated behavioral response (Figure 1). For example, via olfactory receptors, leading to an aversive behavior.

As discussed, microorganisms have been shown to influence decision-making in animals via olfactory processes (Qiao et al., 2019; O’Donnell et al., 2020). In humans, the olfactory system plays a major role in social behavior. For example, olfactory cues can significantly influence memory recall, purchasing behavior, appetite and sexual arousal (Borg et al., 2014; Jacobson et al., 2019; Sandell, 2019). As such, the *Holobiont Blindspot* could potentially have important social ramifications. To illustrate this, we present a brief thought experiment below:

1. Changes to (or inter-individual differences in) the human microbiome via environmental disturbances (e.g., pollution exposure; dietary change; antibiotics) → 2. Changes to odor perception in the human host → 3. Changes to preferences (e.g., human odors as “attractants”) → 4. Could the hypothetical individual become less attracted to another individual as a result of this microbially mediated driver? → 5. Theoretically, this could have important social implications (e.g., leading to relationship issues).

Indeed, it has previously been demonstrated that the microbiome can influence mating preferences in the *D. melanogaster* model. For example, Sharon et al. (2010) divided a population of *D. melanogaster* and reared one half of the population on a molasses-based medium and the other on a starch-based medium. When the populations were mixed together, the flies reared on molasses preferred to mate with other “molasses flies” and the “starch flies” preferred to mate with other starch flies. However, subsequent treatment with antibiotics abolished mating preference in the flies suggesting the microbiome was responsible for the preferences. When the flies were inoculated with microbiota from the media, this phenomena was confirmed. It is thought that the microbiome has a role in changing the levels of sex hormones, thus influencing mating behavior.

Other examples could have important health implications – such as potential effects on food selection or influencing our choices to spend time in certain environments (salutogenic or otherwise). Indeed, the *Lovebug Effect* (Robinson and Breed, 2020) was recently proposed as a microbially mediated mechanism to help explain our affinity for nature – i.e., could a deficiency in the diversity and functional potential of gut microbiota influenced our decision, via the microbiota-gut-brain axis, to spend time in natural environments where immune supporting microorganisms are abundant? The *Umwelt* of an individual is shaped by the environments the individual resides in, and by the interactions they engage in Baggs and Chemero (2019). Therefore, microbial drivers of behavior could profoundly influence the *Umwelt* of the individual.

Our microbiome is also thought to affect our mood (Bastiaanssen et al., 2020; Talbott et al., 2020). Could this have implications for our relationships and motivations, with downstream effects, for example, on work performance and mental health? After all, in the case of depression, oftentimes people do not know (and so cannot articulate) why they feel depressed (Cheng et al., 2020) – could this also be a *Holobiont Blindspot*? If there is a microbial link to depression as suggested by researchers (Foster and Neufeld, 2013), investigating interventions (e.g., through microbial therapeutics) to address this could play an important role in managing mental health in the future (Long-Smith et al., 2020). Several studies have also shown that fecal microbiota transplants can result in the transfer of behavioral phenotypes such as anxiety-like behaviors and anhedonia (inability to feel pleasure) (Bercik et al., 2011; Kelly et al., 2016; Cryan et al., 2019). One study found that altering microbiota in germ-free mice led to changes in hippocampal brain-derived neurotropic factor (a protein involved in brain development and regulation) and subsequent differences in exhibited anxiety-like behaviors (Bercik et al., 2011). Therefore, the *Holobiont Blindspot* could conceivably lead to an inadequate explanation of anxiety-like and anhedonic behaviors, whereas taking host-microbiome interactions into account could provide a much richer and more accurate explanation. Indeed, microbially mediated anhedonic behaviors is another potential pathway to which microbial drivers could affect one’s *Umwelt* i.e., through altering the perception of pleasure.

Our microbial symbionts also affect cognitive traits such as memory, which could affect host memory of food location, as recently shown in wild vertebrates (Davidson et al., 2020). This could have important dietary and health implications, and in humans could conceivably cause relationship issues – e.g., if one partner regularly forgets an important date or forgets to express affection. Further investigations into these theoretical *Holobiont Blindspots* could change the way we understand and empathize with certain social behaviors. As System 1 thinking plays a role in systematic errors through reasoning (Kannengiesser and Gero, 2019; Preisz, 2019), studies aimed at ascertaining the potential effects (deleterious or otherwise) of a host’s microbiome in this process could be extremely valuable. If part of our perception and intuition is influenced by “other” agents (i.e., microorganisms) considered to be constituents of the holobiont, could this change the way we view perception and intuition? Or even the way we view each other – e.g., procuring empathy for decisions “out of our control,” or mitigating intuitions/impulses that lead to unfavorable actions? The *Holobiont Blindspot* could also be related to the psychological model of “free will,” which has implications for the notions of responsibility and punishment. Indeed, alterations to certain regions of the brain such as the prefrontal cortex can “produce an individual capable of differentiating right from wrong but who, nonetheless, is organically incapable of appropriately regulating their behavior” (Zeki et al., 2004, p. 1). Could our microbiome affect our perception-action cycle and System 1 responses via the modulation of irresistible impulses, and should this be taken into account when considering responsibility and the notions of “free will” and determinism?

Following a similar logic to the recently proposed *Sozialwelt*, we argue that more attention should be given to the hidden components of the system that could influence an organism’s *Umwelt* (e.g., the microbiome). As suggested, microorganisms could have an important role to play in the *Umwelt* through perception (*Merkwelt*) and action (*Wirkwelt*). We should be alert to the possibility of a *Holobiont Blindspot* and consider that “thinking” is not simply a brain-centric process – microorganisms may play a role in a complex suite of interactions between the brain, body and environment. Indeed, the *Holobiont Blindspot* and the *Umwelt* are also relevant through the lens of biological individuality. If the *Umwelt* refers to an organism’s perceptual world, and the individuality of an organism is in question – particularly given that holobionts can be considered to be individuals and ecosystems simultaneously (Suárez and Stencel, 2020) – then is the *Umwelt* the perceptual world of an organism or an ecosystem? The *Holobiont Blindspot* questions the very mechanisms and boundaries of the *Umwelt* and even the notions of free will and determinism. It will hopefully generate discussion about how far the microbiome can go in terms of explaining “our” behavior and evolution.

## Conclusion

In this perspective article, we have discussed the importance of considering microbial influences on what is traditionally considered to be an organism’s perceptual world (*Merkwelt*) and action world (*Wirkwelt*) – and in the absence of doing so, there is potential for the *Holobiont Blindspot* (a cognitive bias) which could have important social ramifications. Indeed, it could be important to study the *Holobiont Blindspot* from both the third-person perspective (e.g., a researcher studying animal populations) and from the first-person view (e.g., comprehending the microbiome’s influence on our own intuition/behavioral responses and even our mental health). Recognizing the *Holobiont Blindspot* and investigating how the microbiome may influence the *Umwelt* and cognition, could also provide new and important insights in evolutionary studies of cognition and social behavior. The *Holobiont Blindspot* may inhibit our understanding and appreciation of the complex interrelated ecologies of biology and behavior. The way we think about *how* we think may need to be revisited.

“Beneath our superficial differences we are, all of us, walking communities of bacteria. The world shimmers, a pointillist landscape made of tiny living beings.” (Lynn Margulis, in Margulis and Sagan, 1986, p. 191).

## Author Contributions

JR conceived, designed, and wrote the manuscript, and produced the figure and data visualizations. JR and RC contributed to manuscript internal critical review process and revisions, and read and approved the submitted version. Both authors contributed to the article and approved the submitted version.

## Conflict of Interest

The authors declare that the research was conducted in the absence of any commercial or financial relationships that could be construed as a potential conflict of interest.
